# Comparative transcriptomics of multidrug-resistant *Acinetobacter baumannii* in response to antibiotic treatments

**DOI:** 10.1038/s41598-018-21841-9

**Published:** 2018-02-23

**Authors:** Hao Qin, Norman Wai-Sing Lo, Jacky Loo, Xiao Lin, Aldrin Kay-Yuen Yim, Stephen Kwok-Wing Tsui, Terrence Chi-Kong Lau, Margaret Ip, Ting-Fung Chan

**Affiliations:** 1 School of Life Sciences, The Chinese University of Hong Kong, Hong Kong SAR, China; 2 Partner State Key Laboratory of Agrobiotechnology, The Chinese University of Hong Kong, Hong Kong SAR, China; 3 Department of Microbiology, The Chinese University of Hong Kong, Hong Kong SAR, China; 4 School of Biomedical Sciences, The Chinese University of Hong Kong, Hong Kong SAR, China; 5 Department of Biomedical Sciences, City University of Hong Kong, Hong Kong SAR, China; 6 Present Address: 3D Medicines Corporation, Shanghai, China; 7 Present Address: Washington University School of Medicine, Saint Louis, MO USA

**Keywords:** Gene expression, Transcriptomics, RNA sequencing, Antimicrobial resistance

## Abstract

Multidrug-resistant *Acinetobacter baumannii*, a major hospital-acquired pathogen, is a serious health threat and poses a great challenge to healthcare providers. Although there have been many genomic studies on the evolution and antibiotic resistance of this species, there have been very limited transcriptome studies on its responses to antibiotics. We conducted a comparative transcriptomic study on 12 strains with different growth rates and antibiotic resistance profiles, including 3 fast-growing pan-drug-resistant strains, under separate treatment with 3 antibiotics, namely amikacin, imipenem, and meropenem. We performed deep sequencing using a strand-specific RNA-sequencing protocol, and used *de novo* transcriptome assembly to analyze gene expression in the form of polycistronic transcripts. Our results indicated that genes associated with transposable elements generally showed higher levels of expression under antibiotic-treated conditions, and many of these transposon-associated genes have previously been linked to drug resistance. Using co-expressed transposon genes as markers, we further identified and experimentally validated two novel genes of which overexpression conferred significant increases in amikacin resistance. To the best of our knowledge, this study represents the first comparative transcriptomic analysis of multidrug-resistant *A*. *baumannii* under different antibiotic treatments, and revealed a new relationship between transposons and antibiotic resistance.

## Introduction

*Acinetobacter baumannii* is a nosocomial pathogen that can grow in diverse environments, including intensive care units. Immunocompromised patients are vulnerable to *A*. *baumannii* infection even during therapy. These bacteria enter the body through moist tissues and can colonize various sites, such as the respiratory tract, central nervous system, skin, and eyes. Pneumonia, blood stream infections, and meningitis are the most frequent adverse effects of infection^1^. The major challenge in the treatment of *A*. *baumannii* infection is multidrug resistance. Pan-drug-resistant *A*. *baumannii* strains have been reported across Asia and Europe^2–4^.

Several comparative genomic studies of *A*. *baumannii* have discussed the relationships among the variability of drug resistance islands and drug-resistant phenotypes^2–4^. Multidrug-resistant strains possess a higher metabolic capacity in environments with limited resources^5^. Nonetheless, information from genome sequences is not sufficient to capture the dynamics of gene expression. Several transcriptome studies have probed the transcriptional changes in the *A*. *baumannii* reference strain ATCC17978 when grown under harsh environmental conditions, such as ethanol treatment^6^, high salinity^7^, and iron deficiency^8^. These studies particularly noted the strong survivability of *A*. *baumannii* under such conditions. Recent transcriptome studies have also investigated the response of this species to antibiotics. For example, a study in which the *A*. *baumannii* reference strain ATCC19606 was treated with colistin and doripenem revealed the critical genes involved in the killing process of colistin^9^, while in another study the multidrug-resistant *A*. *baumannii* strain MDR-ZJ was treated with tigecycline and identified an efflux pump and several transcriptional regulators involved in the drug resistance response^10^. These studies were performed using only single strains, which may limit the generalizability of their conclusions. Due to the large variations in genome sizes and sequences among different strains, especially between multidrug-resistant and -sensitive ones, a more systematic analysis that is free from strain bias is needed to study and compare their transcriptomes under antibiotic-free and -treated environments.

Herein, to better understand the transcriptomic response of drug resistance in *A*. *baumannii*, we conducted a comparative study of nine multidrug-resistant strains, five of which are pan-drug-resistant, and three sensitive strains using a strand-specific RNA-sequencing protocol. We adopted a *de novo* transcriptome assembly and analysis pipeline to study gene expression in the form of polycistronic transcripts. Differentially expressed genes were analyzed according to the corresponding strain properties. Three multidrug-resistant strains were further treated with three antibiotics, namely amikacin, imipenem and meropenem, and the corresponding transcriptomes were generated and analyzed. Our results indicated that genes associated with transposable elements in general were up-regulated under antibiotic treatment. Many of these genes are known to be drug resistance-related. Among several previously unknown transposon-associated genes, we experimentally validated two of them. Expression of these two genes in *E*. *coli* BL21 conferred increased resistance to amikacin. In summary, we believe our comparative transcriptomic study and polycistronic transcript analysis revealed new insights into the drug resistance response in *A*. *baumannii*. Our analytical approach should also be applicable to similar studies of other bacterial pathogens.

## Results

### *A*. baumannii strains with different drug resistance profiles exhibited differential growth rates in antibiotic-containing media

**Figure 1:**
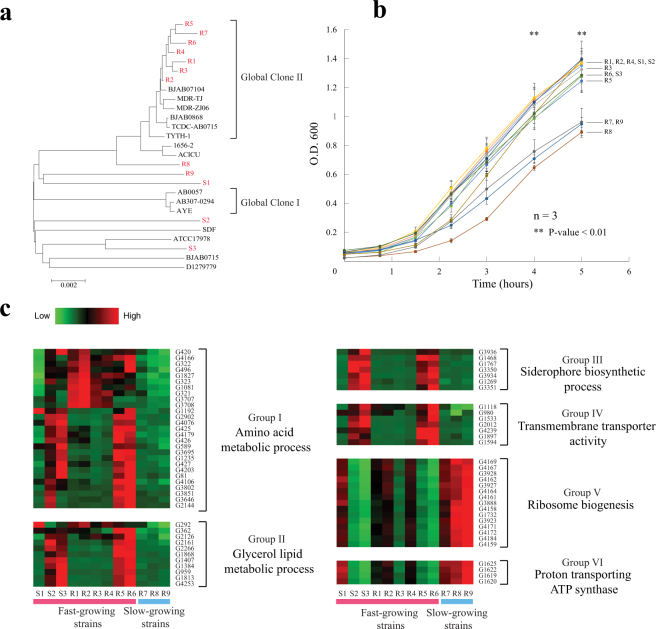
Fast-growing pan-drug-resistant strains exhibited higher nutrition uptake and utilization ability. (**a**) Phylogenetic tree including collected strains and 15 published complete genomes. (**b**) Growth curves of 12 strains in BHI broth over 5 hours. Error bars: standard error. *p*-values were calculated by Student’s *t*-test. (**c**) Heat map of differentially expressed genes between fast-growing strains and slow-growing strains, classified by functional groups.

Twelve strains with different pulsed-field gel electrophoresis (PFGE) patterns (Fig. S1) and antibiotic resistance profiles were collected (Table S1). These include nine multidrug-resistant strains (R1 to R9), five of which are pan-drug-resistant (R1, R5, R6, R7, and R8), and three sensitive strains (S1 to S3) (Table S1). Seven multidrug-resistant strains (R1 to R7) of the Ia PFGE pattern were clustered to the Global Clone II (GC II)^4^ of multidrug-resistant *A*. *baumannii* (Fig. 1a). The other strains belonged to clones other than GC I and GC II. Three multidrug-resistant strains (R7, R8, and R9) grew significantly slower than the other nine strains after 4 hours (Fig. 1b) based on a pooled two-tailed Student’s *t*-test (*p* < 0.01).

### *De novo* transcriptome assembly revealed gene-gene relationships in polycistronic transcripts

All 12 strains were cultured in antibiotic-free conditions until mid-log phase. Three multidrug-resistant strains, R3, R4, and R5, which are from GC II and show similar PFGE patterns, were also cultured in antibiotic-containing media. Three antibiotics, amikacin (an aminoglycoside), imipenem, and meropenem (both of the carbapenem class) were chosen, because the 12 strains had exhibited various degrees of resistance to these three antibiotics. In total, 18 antibiotic-treated samples were prepared from either mid-log or stationary phases, but only 17 samples, excluding the amikacin-treated R4 at mid-log phase, were successfully sequenced. RNA sequencing generated a read depth of over 1,000× per sample. A total of 177,939 transcripts were assembled by Trinity^11^ across 29 samples. 38.8% of the assembled transcripts could be annotated based on known genes from 15 complete *A*. *baumannii* genomes on the transcribing strand, while the other 61.2% of the transcripts could be classified into ERCC spike-in sequences, antisense transcripts, or mis-assembled transcripts. Among these annotated transcripts, over 75% were polycistronic, along which more than one gene could be annotated (Fig. S2). Polycistronic mRNAs are produced by prokaryotes to regulate functionally related genes in operons^12^. In our results, over 97.5% of the genes were annotated from at least one polycistronic transcript (Fig. S3a). Genes that were on the same transcripts, particularly those within three loci of one another, had a higher chance of being annotated under the same gene ontology (GO) hierarchy (biological process) compared with those that were on different transcripts (*p* < 0.01) (Fig. S3b). Many of these pairs of genes encoded on the same transcript had significantly positive Spearman correlations in terms of expression levels. The median Spearman correlation between genes on the same transcripts was significantly greater than 0 (*p* < 0.01) (Fig. S3c).

### Amino acid metabolism and membrane transporters were up-regulated in fast-growing pan-drug-resistant strains

To investigate the major pathways associated with growth rates, the transcriptomes were compared between fast-growing and slow-growing strains at the antibiotic-free mid-log phase. In total, 133 up-regulated and 53 down-regulated genes were identified in fast-growing strains when compared with slow-growing strains. Based on their GO annotations, six GO terms were functionally correlated with the growth rate based on Pearson’s chi-square test (*q* < 0.01) followed by the Benjamini–Hochberg procedure (Fig. 1c). Genes that are involved in amino acid metabolic processes and that exhibited the most expression variability among multidrug-resistant strains were all significantly up-regulated in fast-growing strains. Contrary to the two slow-growing pan-drug-resistant strains R7 and R8, the two fast-growing strains, R5 and R6, exhibited a high degree of similarity in expression patterns to the fast-growing, sensitive strains S2 and S3, especially for genes involved in amino acid metabolism, glycerol lipid metabolism, siderophore biosynthesis, and transmembrane transporters.

### Metabolic pathways centered at and around the TCA cycle were consistently up-regulated in all three antibiotic treatments

**Figure 2:**
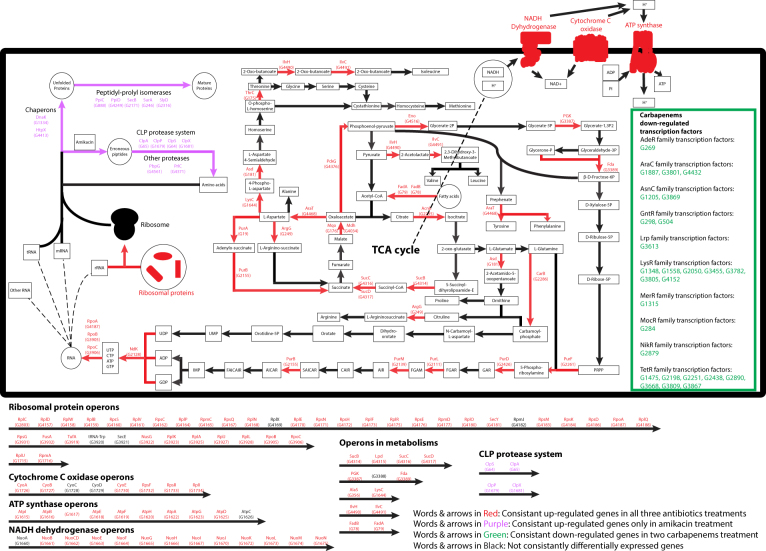
Genes that were differentially expressed under antibiotic treatment. Red genes or arrows: genes that were consistently up-regulated under treatment with all three antibiotics. Purple genes or arrows: genes that were consistently up-regulated only under treatment with amikacin. Green genes or arrows: genes that were consistently down-regulated only under treatment with the two carbapenem-related antibiotics.

To investigate which genes and pathways were significantly modulated under antibiotic treatment, the transcriptomes of the antibiotic-free mid-log phase were compared with those of the antibiotic-treated mid-log phase and antibiotic-treated stationary phase from three multidrug-resistant strains (R3, R4, and R5). A total of 559 genes were up-regulated and 910 genes were down-regulated in treatment with at least one of the three antibiotics (Fig. S4a). Among them, 172 genes were commonly up-regulated in all three antibiotic treatments, with genes involved in metabolism constituting the largest group of these (Fig. 2). Genes coding for enzymes involved in the TCA cycle, and in the biosynthesis of some amino acids, purines, and pyrimidines whose raw materials originate from the TCA cycle, were up-regulated. The complete operons encoding enzyme complexes responsible for ATP synthesis, including NADH dehydrogenase, cytochrome C oxidase, and ATP synthase, and ribosomal protein operons, were also up-regulated.

Antibiotic-specific responses were also studied. The three *A*. *baumannii* strains exhibited distinct responses to the two classes of antibiotics (Fig. S4a). Amikacin specifically induced up-regulation of 149 genes, but only 4 genes were down-regulated. Those up-regulated genes were significantly enriched in unfolded protein binding, protein folding, and proteolysis based on a hypergeometric test of enrichment (*q*-value < 0.01), including two protein-folding chaperons (DnaK and HtpX), several peptidyl-prolyl isomerases, and the CLP protease system (Fig. 2). In contrast, more than 400 genes were commonly down-regulated when treated with either of the two carbapenem-related antibiotics, and only 17 genes were commonly up-regulated. Many transcription factors were commonly down-regulated in treatment with imipenem or meropenem. However, up to 60% of the genes that were commonly down-regulated in carbapenem do not have any GO-term annotation.

The three strains also exhibited strain-specific responses under different antibiotic treatments (Fig. S4b). Many more genes were down-regulated in the pan-drug-resistant strain R5 when compared with R3 and R4. About 24% of these R5-specific down-regulated genes were affected by all three antibiotic treatments, of which many were transmembrane transporters and other membrane proteins involved in signal transductions (Fig. S4c).

### Antibiotic resistance genes were expressed in both antibiotic-resistant and -sensitive strains

**Figure 3:**
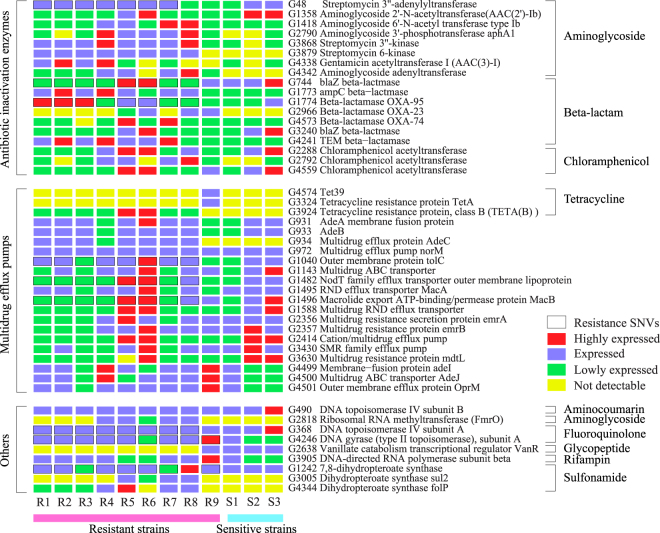
Antibiotic resistance genes were widely distributed in resistant and sensitive strains. Each square represents the expression level of a gene (across the row) in a given strain (down the column). Genes are grouped according to protein functions, followed by the type of antibiotic resistance that the gene is associated with.

The Comprehensive Antibiotic Resistant Database^13^ includes a total of 48 known antibiotic resistance-related genes that were identified in at least one strain (Fig. 3), which include antibiotic inactivation enzymes, multidrug efflux pumps, and direct antibiotic targets. Some of the well-documented resistance-related mutations were also identified in the multidrug-resistant strains, such as the two fluoroquinolone-resistance-conferring mutations: serine-to-leucine at the 83^rd^ residue in the DNA gyrase subunit A, and serine-to-isoleucine at the 80^th^ residue in the DNA topoisomerase IV subunit A^14^. Moreover, the expression patterns of the drug resistance genes attributed to the phenotype of the antibiotic-resistant strains. For example, beta-lactamase which is an enzyme to break down the structure of beta-lactam and multidrug efflux pump which transports the antibiotics out of the cell (Fig. 3) in particular meropenem were highly expressed in R5 and R6 strains (Table S1) and these two strains were resistant to MEM and IPM. Intriguingly, on the other hand, many antibiotic resistance genes were also found to be expressed in the sensitive strains (Fig. 3). On this basis, the presence of antibiotic resistance in the resistant strains may not be solely due to the expression of one particular antibiotic inactivation enzyme or efflux pump. This phenomenon also suggested the combinational and synergic effects of the drug resistance genes expressed and functioned with each other in antibiotic resistant bacteria.

### Transposon-associated antibiotic resistance genes are major contributors to antibiotic resistance

**Figure 4:**
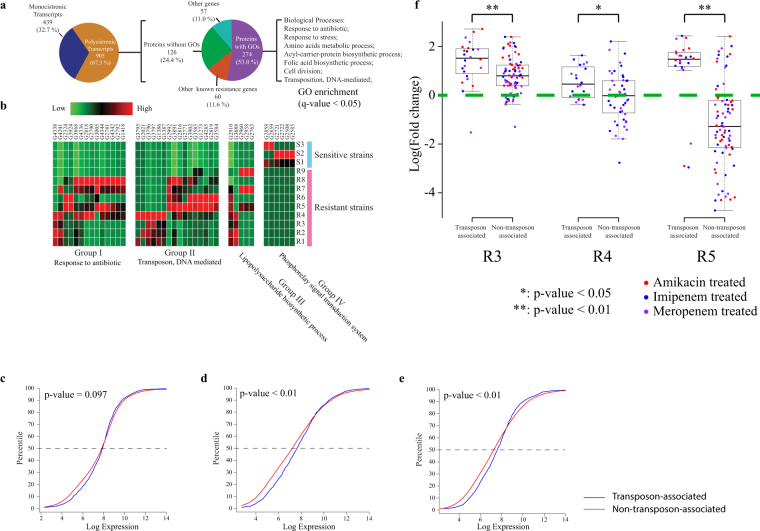
Transposons were up-regulated in multidrug-resistant strains and their associated genes maintained relatively higher expression in antibiotic-treated environments. (**a**) Statistics of genes co-transcribed with known antibiotic resistance genes. (**b**) Heat map of differentially expressed genes between multidrug-resistant strains and -sensitive strains, classified by functional groups. (**c**–**e**) Comparison of percentile-of-expression values between transposon-associated genes and non-transposon-associated genes at antibiotic-free mid-log phase (**c**), antibiotic-treated mid-log phase (**d**), and antibiotic-treated stationary phase (**e**). Blue line represents transposon-associated genes; red line represents non-transposon-associated genes. *p*-values were calculated by Wilcoxon’s rank-sum test. (**f**) Boxplots of fold changes of known antibiotic resistance genes under the antibiotic treatments. Each point represents a log(fold change) value of a known antibiotic resistance gene under a certain antibiotic treatment. *p*-values were calculated by Wilcoxon’s rank-sum test.

To investigate the driving factors, other than the expression of antibiotic resistance genes, of drug resistance, we also examined the extent to which antibiotic resistance genes appeared in polycistronic transcripts. Known antibiotic resistance genes were annotated in over 67% of the polycistronic transcripts (Fig. 4a). Over 50% of the other co-transcribed genes could be annotated based on their GO terms. These genes are enriched in several functions, including amino acid metabolic processes, cell division, and transposition, revealing the complex transcriptional regulation of the antibiotic response, with significant *q*-values as calculated by the Benjamini–Hochberg procedure (*q*-values < 0.05).

To further identify the genes and pathways that contribute the most to antibiotic resistance, differential gene expression analysis was performed between multidrug-resistant and -sensitive strains at the antibiotic-free mid-log phase. Four groups of genes that were differentially expressed between multidrug-resistant and -sensitive strains were, as expected, functionally correlated with antibiotic resistance based on Pearson’s chi-square statistical test (*q* < 0.01) followed by the Benjamini–Hochberg procedure (Fig. 4b). Responses to antibiotics that involve either many known antibiotic resistance genes or DNA-mediated transposons were both up-regulated in multidrug-resistant strains.

Some genes can be assembled with an annotated transposable element on the same transcript, and will be referred to as “transposon-associated” throughout this study. To globally study the difference in expression patterns between transposon-associated genes and non-transposon-associated genes, the cumulative density curves of the expressions of both groups of genes were plotted. Gene expressions of three multidrug-resistant strains, namely R3, R4, and R5, with or without antibiotic treatment, were pooled according to the conditions (antibiotic-free mid-log phase, antibiotic-treated mid-log phase, or antibiotic-treated stationary phase) to be analyzed. The expression distributions of transposon-associated genes did not show significant differences from non-transposon-associated genes at the mid-log phase under antibiotic-free conditions (Fig. 4c). However, transposon-associated genes generally maintained higher expression levels in both mid-log and stationary phases under antibiotic treatment, indicated by the significant shift of the curves to the right (higher expression) below the 70^th^ percentile based on Wilcoxon’s rank-sum test (*p* < 0.01) (Fig. 4d,e). This observation also held true for individual genes when the cumulative density curves were separately plotted with different antibiotic treatments and strains (Fig. S5). To confirm whether antibiotic resistance had a statistically significant correlation with transposon-associated genes by the chi-square test, 39 known drug resistance genes were categorized according to their functions, expression patterns, and their co-transcribed genes (Fig. S6a). Transposable elements showed a higher probability of being co-transcribed with drug inactivation enzymes, and their associated drug resistance genes were more likely to be up-regulated in multidrug-resistant strains, indicated by the significant *p*-values (*p* < 0.01). These findings led to the observation that up-regulated drug resistance genes were preferentially those coding for drug inactivation enzymes (*p* = 0.007) (Fig. S6b–d). It was also noticeable that all transposon-associated up-regulated drug resistance genes were drug inactivation enzymes (Table S7). Furthermore, comparing the expressions of known drug resistance genes between the antibiotic-free mid-log phase and the antibiotic-treated mid-log phase of each of the three multidrug-resistant strains (R3, R4, and R5) showed that under the antibiotic-treated conditions, transposon-associated resistance genes generally exhibited up-regulation compared with antibiotic-free conditions in all three multidrug-resistant strains, with significant *p*-values (*p* < 0.01) based on the two-tailed Student’s *t*-test. They also showed greater up-regulation fold changes than non-transposon-associated antibiotic resistance genes, with significant *p*-values in Wilcoxon’s rank-sum test (*p* < 0.05) (Fig. 4f). However, non-transposon-associated antibiotic resistance genes were not all up-regulated under antibiotic treatment. Their expression patterns were found to be strain-specific.

**Figure 5:**
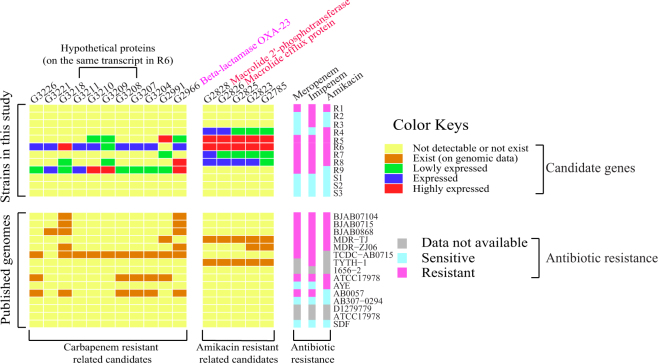
Identification of novel antibiotic-specific resistance genes. Each square represents the expression level or the presence of a gene (down the column) in a given strain (across the row). The resistance of each strain to the three antibiotics is shown on the right panel.

**Figure 6:**
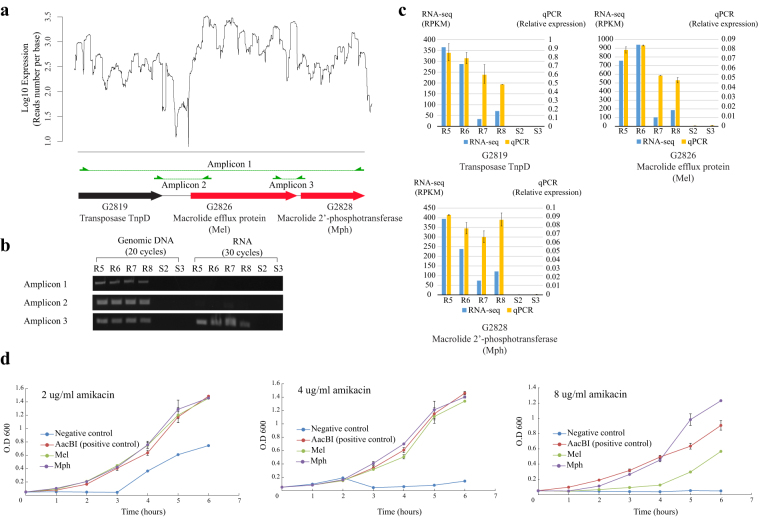
Transposon-associated *mel* and *mph* were co-transcribed and both contributed to amikacin resistance. (**a**) Transcript structure of the transposon gene with the two candidates. (**b**) Reverse-transcription PCR result of connection regions among three genes (*mel*, *mph*, and *tnpD*). (**c**) Quantitative PCR results for those three genes. (**d**) Growth of *E*. *coli* transformed with the two candidate genes (*mel* and *mph*) in different amikacin concentrations.

Based on the drug resistance profiles and transcriptome results of the 12 strains collected in this study, and combined with the 15 published strains with full genome sequences, several candidate resistance genes were identified (Fig. 5). Among these antibiotic-specific resistance genes, beta-lactamase OXA-23 was discovered to be transposon-associated, which was previously reported to confer carbapenem resistance in many carbapenem-resistant strains^3,4,15–18^. In addition, we identified that two transposon-associated genes, namely, *mph*, which codes for the macrolide 2′-phosphotransferase homolog, and *mel*, which codes for the macrolide efflux protein homolog, were assembled onto the same transcript downstream of the transposon gene *tnpD* in all amikacin-resistant strains (R4, R5, R6, R7, and R8), and were also identified in the genomes of *A*. *baumannii* strains MDR-TJ and TYTH-1 (Fig. 6a). This combination of genes can be identified by BLAST on the plasmids in other amikacin-resistant *A*. *baumannii* strains, such as BJAB0714 and BJAB0868, and also in other gram-negative bacteria, such as *Providencia rettgeri*, *Providencia stuatii*, *Klebsiella pneumoniae*, *Klebsiella oxytoca*, *Citrobacter freundii*, *Salmonella enterica*, and *Escherichia coli*, with nearly full-length coverage at >90% identities. It should be noticed that *mph* and *mel* may not be the determinants for amikacin resistance as other antibiotic resistance genes may have an cooperative effect.

### Transposon-associated *mph* and *mel* conferred amikacin resistance in *E*. *coli*

Reverse-transcription PCR (RT-PCR) confirmed that *mph* and *mel* were frequently co-transcribed (Fig. 6b), while *tnpD* and *mel* were also traceably co-transcribed (Fig. S7a). It is noticed that amplicon 1 could not be amplified with RT-PCR, which may indicate an assembly error in this contig. This is the disadvantage of transcriptome assembly using short reads^19^. Quantitative PCR further confirmed that *mel* and *mph* had similar expression levels (Fig. 6c). To study whether the two candidates conferred amikacin resistance, *mel*, *mph* and the positive control *aacBI*, which is known to confer amikacin resistance^20,21^, were independently transformed into the amikacin-sensitive *E*. *coli* BL21 (Table S3). Their protein expressions were confirmed by western blotting (Fig. S7b). Both *mel* and *mph* expression increased the resistance of *BL21* under amikacin treatment at different concentrations (Fig. 6d). The MIC of the negative control was 6 μg/ml, while the *aacBI*, *mel* and *mph* transformed strains had MICs around 16 μg/ml.

## Discussion

Comparative genomics studies have been widely conducted to understand the evolution of pathogens toward multidrug resistance. However, knowledge of the global regulation of antibiotic resistance genes is still inadequate, due to the lack of RNA-seq data and suitable methodologies for comparative transcriptomics. In this study, we collected nine multidrug-resistant and three multidrug-sensitive *A*. *baumannii* strains, cultured them with or without antibiotic treatment, and developed a *de novo* assembly-based method, by which polycistronic transcripts, the major form of transcription in bacteria, could be reconstructed, to analyze the comparative transcriptomics. This novel method of analysis revealed, for the first time, that the regulation of antibiotic resistance genes was performed at the level of operons. Antibiotic resistance genes were systematically analyzed to determine, first, with which genes they were co-transcribed, and second, which genes and pathways were significantly modulated under treatment with antibiotics.

First, our data suggested that under treatment with both classes of antibiotics, genes in several operons that are involved in ATP, RNA, and protein synthesis were simultaneously up-regulated, suggesting that the rates of energy and protein production were generally elevated among multidrug-resistant *A*. *baumannii*. Many genes involved in these pathways were also reported to be up-regulated in *Wolbachia* under doxycycline treatment^22^. Moreover, the strains also responded differently to the two classes of antibiotics. Amikacin, an aminoglycoside that targets the translation machinery, induced up-regulation of over 149 genes, among which were genes involved in protein folding and lysis, while only 4 genes were down-regulated. In contrast, the two carbapenem-related antibiotics (imipenem and meropenem) led to a general down-regulation of gene expression, including of a large number of transcription factors. In addition, most of the imipenem- and meropenem-specific genes encoded either hypothetical proteins or proteins without functional annotation. Thus, there remains a large knowledge gap concerning our understanding of the transcriptional response under carbapenem treatment.

Second, the presence of a single antibiotic resistance gene may not necessarily confer antibiotic resistance in *A*. *baumannii*. A wide spectrum of antibiotic resistance genes have been identified in all strains, including the resistant and sensitive ones (Fig. 3). Some of these genes, e.g. AdeB (G933) (Table S7), a member belonging to the AdeABC efflux pump system, have been reported inadequate in development of aminoglycoside resistance^23^. We have shown in this study that expression of *mel* and *mph* could confer amikacin resistance, though the MIC of *E*. *coli* strains expressing AacBI, Mel and Mph under amikacin were around 16 μg/ml, which was lower than the MIC of the multidrug-resistant *A*. *baumannii* strains (Table S1). Our data suggest that multiple antibiotic resistance genes are necessary to confer antibiotic resistance in clinically isolated multidrug-resistant strains.

Third, our data showed that antibiotic resistance genes that were co-transcribed with transposons generally had higher fold-changes than other resistance genes, which were not associated with transposons, under antibiotic treatment and were more likely to confer antibiotic-specific resistance. Both multidrug-resistant and -sensitive strains possessed a wealth of antibiotic resistance genes. Most antibiotic resistance genes were involved in important operons and were transcribed with other genes. Based on the GO annotations of their co-transcribed genes, antibiotic resistance genes can be classified into two categories: transposon-associated genes, which are co-transcribed with at least one transposon gene, and non-transposon-associated genes. Transposons are among the major players in horizontal gene transfer, which contributes to the evolution of resistance islands in *A*. *baumannii* genomes^24^. It has been reported that the insertion of transposable elements may create active promoters and induce high-level expressions of downstream genes^25,26^. Moreover, our results revealed that transposable elements could also shape gene expression patterns under antibiotic treatment. Not all transposable elements and their associated genes were up-regulated under antibiotic treatment (Fig. S8a,b), but they in general already have higher fold-changes when compared with non-transposon-associated genes. We found that transposon-associated resistance genes had a higher preference to be associated with drug inactivation enzymes, which was also reported recently in a comparative genomic study^2^. Therefore, transposable elements could serve as an efficient marker for identifying co-transcribed candidate antibiotic resistance genes, through which two novel amikacin resistance genes, namely *mel* and *mph*, were reported and experimentally validated in this study. Although the relationships between antibiotic treatment and transcription of resistance genes-associated transposons remains unclear, four proteins have been reported as potential regulators involved in transposons expression^27,28^. In this study, three of these regulators were identified, including FIS (G2401), H-NS (G3933) and IFH (G4261) (Table S8). It would be of interest to investigate relationships between transposons expression and these regulators as it could serve as an effective way of reversing drug resistance by inhibiting the transcriptional activities of transposons.

## Materials and Methods

### Selection of strains

All strains were isolated from patients at hospitals in Hong Kong (Tables S1 and S2). PFGE was performed to identify the strain lineages according to Seifeit *et al*.^29^. The PFGE patterns were clustered using the Unweighted Pair Group Method with Arithmetic Mean. Different lineages were determined by a similarity cutoff of 80%, with 1.5% position tolerance and 1% band optimization. The minimum inhibitory concentrations (MICs) of the antibiotics were determined according to Wiegand *et al*.^30^. Multilocus sequence typing was performed as described at PubMLST (http://pubmlst.org/abaumannii/). Strains with different lineages or antibiotic resistance properties were selected for further study.

### Determination of minimum inhibitory concentration (MIC)

The MIC of the strains was determined and interpreted according to the criteria of Clinical and Laboratory Standards Institute (CLSI)^31^. Briefly, the bacteria were grown in 5% blood agar overnight. Bacterial suspension were prepared to a turbidity equivalent to 0.5 McFarland. Bacteria were inoculated to the antibiotic-containing Mueller-Hinton agar plates and incubated at 37 °C for 16 hours and the MICs were read.

### Bacteria culture

*A*. *baumannii* was first cultured in BHI broth overnight. 1 ml overnight culture was inoculated to 100 ml fresh BHI broth. The mid-log (OD600 ~ 1.00) and stationary (OD600 ~ 2.1) samples were harvested. The bacteria cultures were incubated at 37 °C with 200 rpm shaking.

To determine the optimal antibiotic concentrations for the antibiotic-treated conditions in LB, 2-fold serial dilutions were performed, starting with the MICs as previously determined, to discover the maximum antibiotic concentrations at which bacterial growth was not significantly inhibited compared with that in an antibiotic-free medium. The final antibiotic concentrations used, in a range from 1/8 to 1/64 of the MICs, are listed in Table S4.

### Library preparation and strand-specific RNA-sequencing

Ten to 15 ml of bacteria culture was pelleted by centrifugation at 3,500 *g* for 10 minutes at room temperature. Two to 4 ml of RNAprotect (Qiagen) was added to the pellet and mixed by vortexing. The pellet was re-precipitated by centrifugation at 3,500 *g* for 10 minutes at room temperature, and the supernatant was discarded. Standard TRIzol (Life Technologies) protocol was then applied to extract total RNAs from the cell pellets. To ensure that the DNA was completely removed, DNase digestion was performed and the total RNA samples were further purified by acidic phenol–chloroform. mirVana (Life Technologies) was used to deplete tRNAs and other small RNA portions, and the large RNA molecules were retained. Ribosomal RNAs were then removed by Ribo-Zero rRNA removal kits for gram-negative bacteria (Epicentre). External RNA Controls Consortium (ERCC) RNA Spike-in Control Mixes (Ambion) were added to each rRNA-depleted RNA sample according to the user guide. The sequencing libraries were prepared using a ScriptSeq v2 RNA-seq Library Preparation kit (Epicentre) with rRNA-depleted samples, and all of the libraries were sequenced by Illumina HiSeq 2000 following the strand-specific sequencing protocol for 100 cycles. All RNA-seq data have been deposited to SRA with the accession SRP075337.

### Reads processing and expression calculation

The first six bases of reads and the adaptors were first removed by in-house-developed pipelines. Then, low-quality sequences were trimmed by Trimmomatic 0.30^32^ with the following parameters: SLIDINGWINDOW:4:20 and MINLEN:50.

The transcripts were assembled using Trinity r2013-08-14^11^ with default parameters and specifying–SS_lib_type as FR. All transcripts were annotated using BLAST based on the 15 full *A*. *baumannii* genomes retrieved from the NCBI (Table S5), with both coverage and identity larger than or equal to 0.8. Overlapped annotations on transcripts were further combined if they overlapped each other by at least 70% of their lengths. Based on the gene annotations of the transcripts, the RPKM (reads per kilobase per million mapped reads) values^33^ were calculated to determine the gene expression levels. The expression values were normalized by methods previously described^34^, which can also be found in the supporting documents, under headings Angular based linear regression, Finding the best regression model and Normalizing samples in different conditions. The expression levels of all genes are listed in Table S8.

### Differential expression determination

Differentially expressed genes between two phenotypical groups of strains (fast-growing vs slow-growing, resistant vs sensitive) were determined by a spatial angular method (refer to the Supplementary methods for a detailed description). Differentially expressed genes between antibiotic-free conditions and antibiotic-treated conditions were determined by the following set of criteria: (1) to be considered as an up-regulation under antibiotic treatment, the normalized expression value of the gene in the treated sample at mid-log phase must be larger than or equal to 50 RPKM; and to be considered as a down-regulation, the normalized expression value of the gene in the untreated sample at mid-log phase must be larger than or equal to 50 RPKM; (2) for up-regulation under antibiotic treatment, the normalized expression value of the gene in the treated sample at mid-log phase must be at least 2-fold larger than that in the untreated sample; for down-regulation under antibiotic treatment, the normalized expression value of the gene in the untreated sample at mid-log phase must be at least 2-fold larger than that in the treated sample; (3) finally, for up-regulation under antibiotic treatment, according to (2), the normalized expression value of the gene in the treated sample at stationary phase must be larger than that in the untreated sample at mid-log phase; and for down-regulation, according to (2), the normalized expression value of the gene in the untreated sample at mid-log phase must be larger than that that in the treated sample at stationary phase. Any gene that was up-regulated in at least two out of three strains and at the same time did not show any down-regulation was considered a common up-regulated gene (and conversely for the common down-regulated genes). However, because the amikacin-treated R4 sample at mid-log phase had failed to be sequenced, only genes up-regulated or down-regulated in both the R3 and R5 strains were used to determine common differentially expressed genes for amikacin. For Fig. S5A, the criteria used for the amikacin-treated R4 samples were loosened. Genes whose normalized expression values in the untreated sample at mid-log phase were smaller than those in the treated sample at stationary phase were considered to be up-regulated (likewise, down-regulated genes were considered to be those whose normalized expression values in the treated sample at stationary phase were smaller than those in the untreated sample at mid-log phase).

### Other bioinformatic analysis

Details of the expression normalization are described in the supporting documents, under headings Angular based linear regression, Finding the best regression model and Normalizing samples in different conditions. For phylogenetic analysis, genes that were expressed in the antibiotic-free samples of all strains and could be identified in all 15 of the complete *A*. *baumannii* genomes were selected, whereas transposon-related genes and common drug resistance genes were excluded. The sequences were aligned separately using MUSCLE^35^ 3.8.31 and only the aligned regions were then extracted and concatenated. The phylogenetic trees were constructed using the neighbor-joining method in MEGA 6^36^. To annotate GO terms to each gene, BLASTX searches for all of the genes were performed against the nr protein database with default parameters, and only the top 50 hits were retained, which were then annotated by Blast2GO^37^ with default parameters. GO enrichment was measured in terms of *q*-values, calculated based on a hypergeometric distribution and the Benjamini–Hochberg procedure. The *q*-values of the functional correlation of the GOs were determined using a chi-square distribution in a contingency table and with the Benjamini–Hochberg procedure.

### Validation of differentially expressed genes by quantitative PCR (qPCR)

Power SYBR Green PCR master mix (Life Technologies) was used according to the standard protocol. A master mix solution was made for each sample. Each 20 μl technical replicate contained 5 ng templates, 375 nM primers (Table S6), and 1× power SYBR Green PCR master Mix. qPCR was performed using an ABI 7500 fast qPCR machine with 40 thermal cycles. Each thermal cycle began with a holding stage at 50 °C for 2 minutes, following by another holding stage at 95 °C for 10 minutes. Then, the cycling stage started with a denaturing step at 95 °C for 15 seconds, followed by a 1-minute annealing and elongation step at 60 °C. The top 3 most stably expressed genes at mid-log phase growing in both the antibiotic-free and antibiotic-treated media were used separately as reference genes. ATP-dependent protease (G707) had the smallest variations between biological replications and fitted the trend of RNA-seq. It was therefore chosen as the reference gene in the qPCR validation step.

### First-strand cDNA synthesis

The SuperScript III first-strand synthesis SuperMix for qRT-PCR (Life Technologies) was used. The reaction mixture was prepared according to the standard protocol. The mixture was first incubated at 25 °C for 10 minutes, followed by incubation at 50 °C for 30 minutes. The reaction was terminated at 85 °C for 5 minutes and cooled over ice. 2 U of *E*. *coli* RNase H was added to the mixture and incubated at 37 °C for 20 minutes.

### PCR and Sanger sequencing

A 50-μl reaction mixture was prepared for each PCR reaction, including 45 μl Platinum PCR SuperMix (Life Technologies) solution, 5 μM primers (Table S6) and 5 ng templates. The reaction started with a 2-minute incubation at 94 °C. Twenty cycles were performed with 30 seconds of denaturing at 94 °C, 30 seconds of annealing at 57 °C, and 1 minute/kb extension at 72 °C. The PCR products were purified by PureLink Quick Gel Extraction and a PCR purification Combo Kit (Life Technologies) based on the standard protocol. The correct sequences of the PCR products were confirmed by Sanger sequencing.

### Bacterial transformation

All plasmids were constructed either using a Champion™ pET101 Directional TOPO® Expression Kit following the standard protocol, or by a gene synthesis service from GenScript (Nanjing, China) on pET15b, which is the closest plasmid to pET101 in the sequence (Table S3). 1 to 10 ng plasmids were transformed to BL21 competent cells (Life Technologies) following the standard protocol. Single colonies were selected from the LB agar plate with 50 ug/ml ampicillin. The DNA of the plasmids was extracted and the correct sequences were confirmed by Sanger sequencing.

### Western blot analysis

Bacterial cells were harvested after 4 hours induction in 1 mM IPTG. The OD600 values were adjusted to 1.0. All samples were lyzed in bacterial lysis buffer (1% SDS in PBS) and loaded onto 12% SDS-PAGE for western blot analysis. 1:3000 anti-HIS antibodies (GE 27-4710-01) and 1:2000 anti-FLAG antibodies (Sigma F3165) in 5% fat-free milk were used as primary antibodies to detect the expression of the candidate resistance genes, engineered with either an HIS-tag (for AacBI and Mel) or a FLAG-tag (for Mph).

### Testing of candidate antibiotic resistance genes

Bacteria transformed with the expression vector were incubated at 37 °C in LB at 150 rpm until OD_600_ was equal to 0.4. 1 mM IPTG was added and the bacteria were incubated for 4 hours to induce protein expression. The culture was diluted to a final OD_600_ of 0.01. Amikacin at the desired concentrations along with 1 mM IPTG were added, and the bacteria were incubated at 37 °C and 150 rpm. The OD_600_ values were taken every one hour.

### Electronic supplementary material

Supplementary methods

Supplementary figures

Supplementary tables 1–7

Supplementary table 8
