# Supplementary material for: Comparative transcriptomics of multidrug-resistant Acinetobacter baumannii in response to antibiotic treatments
Source: Sci Rep. 2018 Feb 23;8:3515. doi: 10.1038/s41598-018-21841-9 (PMC5824817; doi:10.1038/s41598-018-21841-9)
Supplement: Supplementary file 1 — Supplementary methods [file 41598_2018_21841_MOESM1_ESM.pdf]

Comparative transcriptomics of multidrug-resistant *Acinetobacter baumannii* in response to antibiotic treatments

Hao Qin[1,2]*, Norman Wai-Sing Lo[3], Jacky Fong-Chuen Loo[1], Xiao Lin[1,2], Aldrin Kay-Yuen Yim[1,2]*, Stephen Kwok-Wing Tsui[4], Terrence Chi-Kong Lau[5], Margaret Ip[3], and Ting-Fung Chan[1,2]#

[1]School of Life Sciences, The Chinese University of Hong Kong, Hong Kong SAR, China

[2]Partner State Key Laboratory of Agrobiotechnology, The Chinese University of Hong Kong, Hong Kong SAR, China

[3]Department of Microbiology, The Chinese University of Hong Kong, Hong Kong SAR, China

[4]School of Biomedical Sciences, The Chinese University of Hong Kong, Hong Kong SAR, China

[5]Department of Biomedical Sciences, City University of Hong Kong, Hong Kong SAR, China

#Address correspondence to Ting-Fung Chan, tf.chan@cuhk.edu.hk.

*Present address: Hao Qin, 3D Medicines Corporation, Shang Hai, China; Aldrin Kay-Yuen Yim, Washington University School of Medicine, Saint Louis, MO, USA.

N.W.S.L and J.F.C.L contributed equally to this work.

Supplementary Methods

Angular based linear regression.

Variable symbol	Variable explanation
$\boldsymbol{n}$	Total number of genes
$\boldsymbol{m}$	Total number of samples
$\boldsymbol{v}$	Raw expression of a certain gene in a certain sample
$\vec{\boldsymbol{v}}_{\boldsymbol{i}}$	Gene vector of gene $\boldsymbol{i}$ constructed by the expression of samples
$\vec{\boldsymbol{r}}$	Reference vector constructed by the size factors of samples
$\boldsymbol{L}$	The sum of cosine values of the angles between the gene vectors and the reference vector
$\boldsymbol{R}$	Raw size factors of samples
$\boldsymbol{s}$	Final size factors of samples

Angular based linear regression method assumes that in a pool of samples, there is a set of house-keeping genes, whose expressions are stable across samples. Due to biological variations, the expressions of a single gene might vary significantly revealed by the fold changes. But the overall variations are majorly contributed by the sequencing process, which are going to be normalized by this method. Finding the overall trend of variations can be approximated by least angle linear regression. Least angle linear regression calculates a line minimizing the sum of angles between the line and the gene vectors in an $\boldsymbol{m}$-dimensional space constructed by the expressions of $\boldsymbol{n}$ genes from $\boldsymbol{m}$ samples. The angles directly indicate the variations of genes' expressions. RNA-seq

measured expressions tend to have larger standard deviations when the absolute magnitudes are large. Least-square regression assumes the standard deviations are the same for all the data points, which can skew the regression line in RNA-seq. Hence considering the angle between the data points and the regression line as error model is more appropriate.

Assuming the vector of gene $\boldsymbol{i}$ is $\vec{\boldsymbol{v}}_{\boldsymbol{i}}$, which is constructed by the expressions $\boldsymbol{v}$ of this gene in all samples. Least angle regression minimizes the sum of angles between gene vectors $\vec{\boldsymbol{v}}_{\boldsymbol{i}}$ and reference vector $\vec{\boldsymbol{r}}$, which is the unit vector representing the regression line. There are many ways to find the least angle regression line. Maximizing the sum of cosine values of the angles is an easy method, because cosine function is strictly monotonic when the angle is between 0° and 180°. The regression line can be approximated by this equation:

$$\text{The sum of angles} \sim L = \sum_{i=1}^{n} \frac{\vec{r} \cdot \vec{v}_i}{|\vec{v}_i|} \qquad (1)$$

where

$$\vec{r} = \begin{bmatrix} r_1 \\ r_2 \\ \vdots \\ r_m \end{bmatrix}$$

$$\sum_{i=1}^{m} r_i^2 = 1$$

The regression line, or the regression vector, generates the maximum value of function $\boldsymbol{L}$. To find the extreme values, the method of Lagrange multipliers is applied:

$$\bigwedge(r_1, r_2 \dots r_m, \lambda) = L(r_1, r_2 \dots r_m) + \lambda(r_1^2 + r_2^2 + \cdots + r_m^2 - 1) \qquad (2)$$

The partial derivatives are set to zero to find the extreme values:

From sample 1 to sample *m*:

$$\frac{\partial \Lambda}{\partial r_i} = 0$$

And:

$$\frac{\partial \Lambda}{\partial \lambda} = 0$$

There is only one extreme value in function $\boldsymbol{L}$, which yields the maximum value. The vector of the regression line actually equals to the sum of all unit gene vectors, which can be calculated by:

$$R_k = \sum_{i=1}^{n} \frac{v_{ik}}{|\vec{v}_i|}, \qquad where\ \ k\ \in [1, m] \qquad (3)$$

The size factor of a certain sample $\boldsymbol{s_k}$ can thus be calculated as:

$$s_k = \frac{R_k}{|\vec{R}|} \cdot \sqrt{m} \qquad (4)$$

After getting the size factors, the normalization can be achieved by a single step:

$$Normalized\ \ expression = \frac{Raw\ \ expression}{Size\ \ factor}$$

Finding the best regression model.

Variable symbol	Variable explanation
$\boldsymbol{w}$	Normalized expression
$\boldsymbol{u}$	Re-scaled normalized expression
$\boldsymbol{\mu}$	Mean of all expression
$\boldsymbol{\sigma}$	Standard deviation of all expressions
$\boldsymbol{P}$	Probability of a certain gene under given model

The most critical process in angular based linear regression method is to determine the set of house-keeping genes. It could be determined based on existing knowledge, which is commonly not available. Hence to tackle the problem, identifying the best set of genes in normalization is another direction, which is only based on the RNA-seq results.

A way to approach the best set of house-keeping genes is using a probabilistic model based on Gaussian distribution. This method calculates a likelihood to indicate the goodness of the model. In this model, house-keeping genes are considered following the Gaussian distribution, while non-house-keeping genes are considered skewed and thus becoming outliers. The best model has the largest likelihood.

Assuming that the normalized expression of gene $\boldsymbol{i}$ in sample $\boldsymbol{k}$ is $\boldsymbol{w_{ik}}$, all the normalized expressions should be re-scaled to $\boldsymbol{u_{ik}}$ by the following calculation to set the equal weight to every gene:

$$u_{ik} = \frac{w_{ik}}{\bar{w}_i} \qquad (5)$$

where $\boldsymbol{\bar{w}_i}$ is the mean of the normalized expressions of gene $\boldsymbol{i}$. Afterwards all the expressions are pooled. The mean $\boldsymbol{\mu}$ and standard deviation $\boldsymbol{\sigma}$ are calculated by:

$$\mu = \frac{1}{m \times n} \cdot \sum_{i=1}^{n} \sum_{k=1}^{m} u_{ik}$$

$$\sigma = \sqrt{\frac{\sum_{i=1}^{n} \sum_{k=1}^{m} (u_{ik} - \mu)^2}{m \times n - 1}}$$

Under the size factors calculated by the set of house-keeping genes, the likelihood can be calculated by:

$$\text{Likelihood} = \sum_{i=1}^{n} log_{10}(\prod_{k=1}^{m} P_{ik}) \qquad (6)$$

Where the probability $\boldsymbol{P_{ik}}$ of gene $\boldsymbol{i}$ in sample $\boldsymbol{k}$ is:

$$P_{ik} = \begin{cases} \Phi\left(-\left|\frac{u_{ik}-\mu}{\sigma}\right|\right) \times 2, & if\ i\ belongs\ to\ housekeeping\ genes. \\ 1-\Phi\left(-\left|\frac{u_{ik}-\mu}{\sigma}\right|\right) \times 2, & if\ i\ doesn't\ belong\ to\ housekeeping\ genes. \end{cases}$$

where $\boldsymbol{\Phi}$ is the cumulative function of Gaussian distribution.

The simplest algorithm to find the best set of house-keeping genes is to calculate the likelihoods of all combinations. However it is resource and time consuming, which could be impossible when the number of genes is huge. Therefore better algorithm is indispensable to reduce the combinations to be calculated. One strategy is to find the best number of house-keeping genes, and then refine the combination of the house-keeping gene set around the number. Firstly to find the best number of house-keeping genes, the initial size factors are calculated with the whole set of genes. Then in each round, the gene, which has the smallest likelihood in the house-keeping gene set of last round, is eliminated from the house-keeping gene set and the new initial size factors are calculated based on the new set of house-keeping genes, until all the genes are eliminated from the house-keeping gene set. The number of the house-keeping genes, which has the maximum likelihood in this step, is selected. Secondly, to further refine the house-keeping gene set, starting from the best gene set in the last step, the state of the gene with the smallest probability is replaced by its opposite state, which means that if the gene is in the house-keeping gene set, it is eliminated and vice versa. Then

the likelihood of the new set of house-keeping genes is calculated. If the new likelihood is larger than the old likelihood, this step will be repeated until the new likelihood is not larger anymore. After this step, the best set of house-keeping genes is determined and chosen in the normalization.

Normalizing samples in different conditions. In this study, different samples have various conditions. Between conditions different sets of house-keeping genes should be assumed. Therefore the size factors should be calculated in a progressive process. This process includes two steps. Firstly, the condition which has the most number of samples is chosen as the base pool to set the standard scale, and the size factors $\boldsymbol{s}$ of these samples are calculated. In this study, the samples in antibiotic-free medium harvested at mid-log phase are normalized first. Secondly, relative to a certain sample $\boldsymbol{q}$ in the base pool, the samples which are conditionally related to sample $\boldsymbol{q}$ are pooled accordingly and their relative size factors in the new scale are calculated. In the third step, the relative size factors can be converted to standard scale through the relative size factor of sample $\boldsymbol{q}$ in the new scale:

$$s_k = \frac{s'_k \times s_q}{s'_q} \qquad (7)$$

where $\boldsymbol{k}$ is the sample pooled in the new scale, which is needed to be converted to standard scale, $\boldsymbol{s}$ is the size factor in the standard scale and $\boldsymbol{s}'$ is the size factor in the new scale. In this study, all antibiotic-treated samples harvested at mid-log phase were grouped with the normal mid-log phase samples accordingly and their size factors in the standard scale were calculated. At last repeat the step 2 and step 3 to the rest of the

samples until all size factors in standard scales are calculated. In this study, the samples treated by antibiotics and harvested at stationary phase are grouped with the samples treated by antibiotics and harvested at mid-log phase accordingly. There is a special case. Since the mid-log phase sample of R4 treated by amikacin is missing, the stationary phase sample of R4 treated by amikacin was grouped with the stationary phase sample of R5 treated by amikacin, which is also an amikacin resistant strain.

Prioritizing differential genes in two pooled groups.

**Variable symbol**	**Variable explanation**
$\boldsymbol{w}$	Normalized expression
$\vec{\boldsymbol{v}}$	Gene vector constructed by the expressions of a certain gene in all samples
$\vec{\boldsymbol{A}}$	The trend vector where the genes are prioritized
$\vec{\boldsymbol{B}}$	The opposite trend vector
$\boldsymbol{S}$	The subspace constructed by $\vec{\boldsymbol{A}}$ and $\vec{\boldsymbol{B}}$
$\boldsymbol{c}$	The coordinate of a certain gene in the subspace $\boldsymbol{S}$
$\boldsymbol{\theta}$	The angle between trend vector $\vec{\boldsymbol{A}}$ and the projected gene vector in subspace $\boldsymbol{S}$
$\vec{\boldsymbol{N}}$	The central vector in the subspace constructed by the samples in group **A**
$\boldsymbol{\varphi}$	The error angle between the central vector $\vec{\boldsymbol{N}}$ and the projected gene vector in subspace constructed by the

	samples in group **A**
$\boldsymbol{\Theta}$	The sorting parameter

It is common to sort the genes that are differentially expressed in two groups. In this study, the genes that are differentially expressed between two groups, multidrug resistant strains and drug sensitive strains, are targets to be further analyzed. Each strains are biologically different and grouped. Two groups are labeled as **A** and **B**, whose number of samples are $\boldsymbol{t_A}$ and $\boldsymbol{t_B}$ respectively. In an $\boldsymbol{m} = \boldsymbol{t_A} + \boldsymbol{t_B}$ dimensional space, all genes, whose number is $\boldsymbol{n}$**,** become vectors, constructed by their expression $\boldsymbol{w_i}$ and annotated by $\vec{\boldsymbol{v}}_{\boldsymbol{i}}$ for gene $\boldsymbol{i}$:

$$\vec{v}_i = \begin{bmatrix} w_{iA1} \\ w_{iA2} \\ \vdots \\ w_{iAt_A} \\ w_{iB1} \\ w_{iB2} \\ \vdots \\ w_{iBt_B} \end{bmatrix}, where \ \ i \in [1, n]$$

If the genes need to be prioritized by the trend where the genes are only expressed in samples of group **A**. The trend vector $\vec{\boldsymbol{A}}$ is defined as:

$$\vec{A} = \begin{bmatrix} \left.\begin{matrix} 1 \\ \vdots \\ 1 \end{matrix}\right\} & t_A \\ \left.\begin{matrix} 0 \\ \vdots \\ 0 \end{matrix}\right\} & t_B \end{bmatrix}$$

Similarly, the opposite trend of $\vec{\boldsymbol{A}}$ is annotated by $\vec{\boldsymbol{B}}$, where the genes are only expressed in samples of group **B**. It is defined as:

$$\vec{B} = \begin{bmatrix} \left.\begin{matrix} 0 \\ \vdots \\ 0 \end{matrix}\right\} & t_A \\ \left.\begin{matrix} 1 \\ \vdots \\ 1 \end{matrix}\right\} & t_B \end{bmatrix}$$

$\vec{\boldsymbol{A}}$ and $\vec{\boldsymbol{B}}$ construct a sub-space $\boldsymbol{S}$ = span$\{\vec{A}, \vec{B}\}$ in the $\boldsymbol{m}$ dimensional space, where $\vec{\boldsymbol{A}}$ and $\vec{\boldsymbol{B}}$ are a pair of orthogonal basis. Each gene in the $\boldsymbol{m}$ dimensional space can be projected to the sub-space $\boldsymbol{S}$. For a gene $\boldsymbol{i}$, the coordinates in $\boldsymbol{S}$ can be converted by:

$$c_A = \frac{\vec{v}_i \cdot \vec{A}}{\left|\vec{A}\right|^2}, c_B = \frac{\vec{v}_i \cdot \vec{B}}{\left|\vec{B}\right|^2} \qquad (8)$$

To calculate the prioritizing parameter for a group, the reference line is first set to the trend vector of the group. For example, the genes are going to be prioritized according to trend $\vec{\boldsymbol{A}}$. The angle $\boldsymbol{\theta}$ between the projection of gene vector $\vec{\boldsymbol{v}}_{\boldsymbol{i}}$ in subspace $\boldsymbol{S}$ and $\vec{\boldsymbol{A}}$ can measure the extent of differential expression of gene $\boldsymbol{i}$ between group **A** and group **B**. If $\boldsymbol{\theta}$ is close to 0°, the gene $\boldsymbol{i}$ has higher expressions in group **A**. If $\boldsymbol{\theta}$ is close to 90°, the gene $\boldsymbol{i}$ has higher expressions in group **B**. If $\boldsymbol{\theta}$ is close to 45°, the gene $\boldsymbol{i}$ has similar expression levels between group **A** and group **B**. Several trigonometric functions can imply the magnitude of $\boldsymbol{\theta}$, which can be calculated by:

$$\sin\theta = \frac{c_B}{\sqrt{c_A^2 + c_B^2}} \qquad (9)$$

Nevertheless, the projection process disregards the variations within groups, causing ties in the ranking when two or more genes have the same number difference of samples that express the gene between the two groups. The ties can be further broken by integrating an error angle $\boldsymbol{\varphi}$ to the $\boldsymbol{\theta}$. Supposing the genes are sorted to the trend $\vec{\boldsymbol{A}}$, the error angle is calculated in the sub-space constructed by all the expressions of

samples in group **A**. The error angle $\boldsymbol{\varphi}$ is the angle between the projected gene vector $\mathbf{proj}_{\boldsymbol{A}}\boldsymbol{v_i}$ in the sub-space of group **A** and the central vector $\vec{\boldsymbol{N}}$.

$$\cos\varphi = \frac{\text{proj}_A v_i \cdot \vec{N}}{|\text{proj}_A v_i| \cdot |\vec{N}|}, \text{where } \text{proj}_A v_i = \begin{bmatrix} w_{iA1} \\ \vdots \\ w_{iAt_A} \end{bmatrix}, \vec{N} = \begin{bmatrix} 1 \\ \vdots \\ 1 \end{bmatrix} \qquad (10)$$

This equation can be further expanded to:

$$\cos\varphi = \frac{\sum_{j=1}^{t_A} w_{iAj}}{\sqrt{\sum_{j=1}^{t_A} w_{iAj}^2} \cdot \sqrt{t_A}}$$

$$\cos\varphi = \sqrt{\frac{\sum_{j=1}^{t_A} w_{iAj}^2 + 2(w_{iA1} \cdot w_{iA2} + \cdots + w_{iAa} \cdot w_{iAb} + \cdots w_{iA(t_A-1)} \cdot w_{iAt_A})}{(\sum_{j=1}^{t_A} w_{iAj}^2) \cdot t_A}}$$

$$\cos\varphi = \sqrt{\frac{1}{t_A} + \frac{2(w_{iA1} \cdot w_{iA2} + \cdots + w_{iAa} \cdot w_{iAb} + \cdots w_{iA(t_A-1)} \cdot w_{iAt_A})}{(\sum_{j=1}^{t_A} w_{iAj}^2) \cdot t_A}} \qquad (11)$$

where:

$$a, b \in [1, t_A], \qquad a \neq b$$

$$w_{iAj} \geq 0, \qquad j \in [1, t_A]$$

$$\sum_{j=1}^{t_A} w_{iAj}^2 > 0$$

The maximum angle of $\boldsymbol{\varphi}$ is 90° because:

$$\cos\varphi \geq \sqrt{\frac{1}{t_A}}$$

$$\min\cos\varphi = \lim_{t_A \to +\infty} \sqrt{\frac{1}{t_A}} = 0$$

$$\max\varphi = 90^\circ$$

When a certain gene $\boldsymbol{i}$ is only expressed in the samples of group **A**, even only in one sample, the gene should always be considered differentially expressed in group A,

which means that the prioritization angle should be less than 45°. To accommodate that criterion, the sorting parameter is finally calculated by:

$$\text{Sorting parameter} \ \ \sin\Theta = \ \sin(\theta + \frac{\varphi}{2}) \qquad (12)$$

The range of $\boldsymbol{\Theta}$ is [0°,135°], where the range of $\sin\Theta$ is [0,1]. This function is strictly monotonic increasing in the range [0, 0.707], whose angle ranges in the range [0, 45°]. If the case, where only one sample in the group expresses a certain gene while none of the sample in the other group expresses the gene, is considered the minimum criterion to considered an up-regulation, a cutoff value of $\sin\Theta$ can be determined by:

$$\text{Cutoff value} = \sqrt{\frac{1}{2} - \frac{1}{2\sqrt{n}}} \qquad (13)$$

To sort the genes following the trend $\vec{\boldsymbol{B}}$, the sorting parameter and the cutoff value should be calculated using $\vec{\boldsymbol{B}}$ as the reference trend.
