# Supplementary material for: Comparative transcriptomics of multidrug-resistant Acinetobacter baumannii in response to antibiotic treatments
Source: Sci Rep. 2018 Feb 23;8:3515. doi: 10.1038/s41598-018-21841-9 (PMC5824817; doi:10.1038/s41598-018-21841-9)
Supplement: Supplementary file 2 — Supplementary figures [file 41598_2018_21841_MOESM2_ESM.pdf]

Comparative transcriptomics of multidrug-resistant *Acinetobacter baumannii* in response to antibiotic treatments

Hao Qin[1,2]*, Norman Wai-Sing Lo[3], Jacky Fong-Chuen Loo[1], Xiao Lin[1,2], Aldrin Kay-Yuen Yim[1,2]*, Stephen Kwok-Wing Tsui[4], Terrence Chi-Kong Lau[5], Margaret Ip[3], and Ting-Fung Chan[1,2]#

[1]School of Life Sciences, The Chinese University of Hong Kong, Hong Kong SAR, China

[2]Partner State Key Laboratory of Agrobiotechnology, The Chinese University of Hong Kong, Hong Kong SAR, China

[3]Department of Microbiology, The Chinese University of Hong Kong, Hong Kong SAR, China

[4]School of Biomedical Sciences, The Chinese University of Hong Kong, Hong Kong SAR, China

[5]Department of Biomedical Sciences, City University of Hong Kong, Hong Kong SAR, China

#Address correspondence to Ting-Fung Chan, tf.chan@cuhk.edu.hk.

*Present address: Hao Qin, 3D Medicines Corporation, Shang Hai, China; Aldrin Kay-Yuen Yim, Washington University School of Medicine, Saint Louis, MO, USA.

N.W.S.L and J.F.C.L contributed equally to this work.

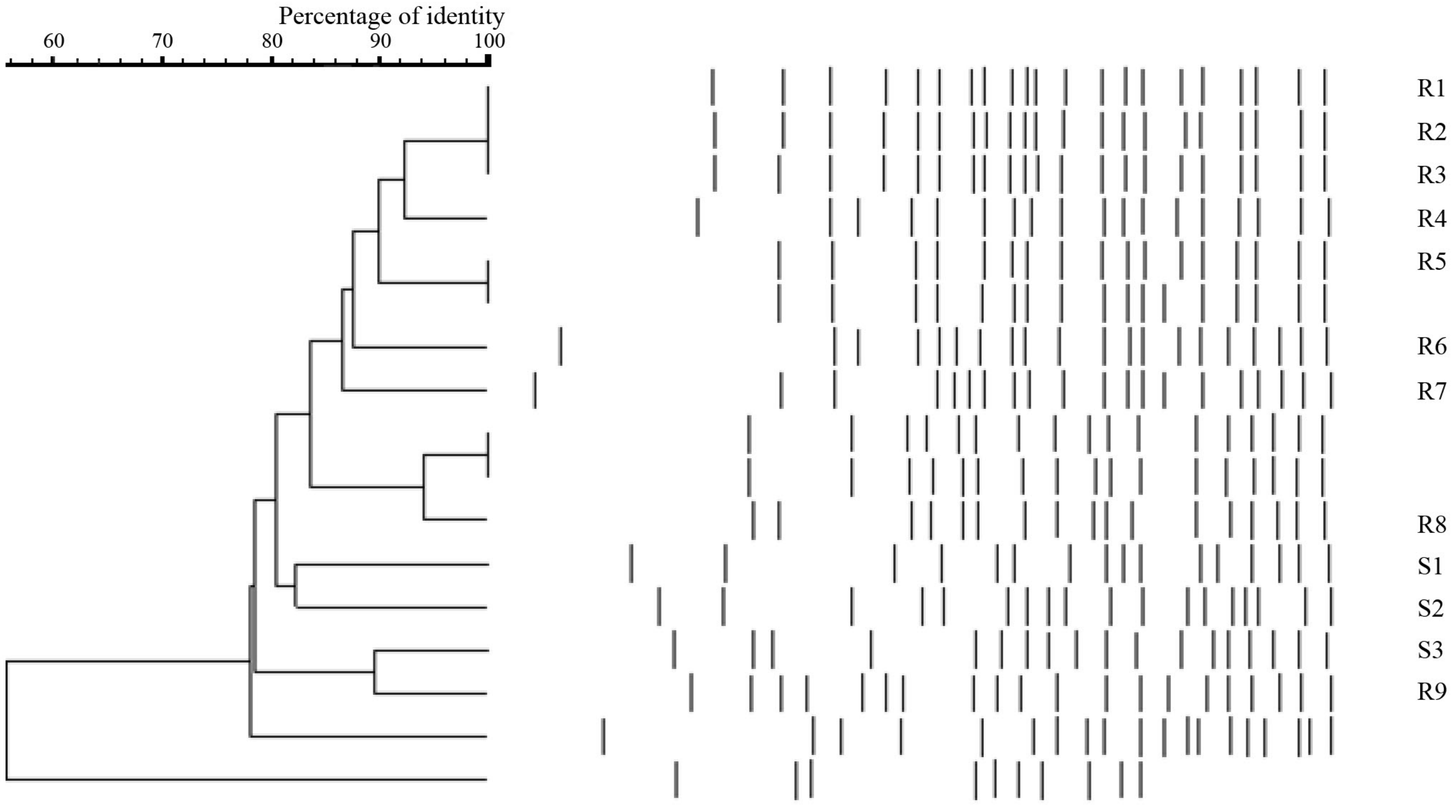

Figure S1. Pulsed-field gel electrophoresis (PFGE) patterns of the A. baumannii strains used in this study. PFGE patterns were clustered using Unweighted Pair Group Method with Arithmetic Mean (UPGMA). Different lineages were determined by the similarity cutoff of 80%, with 1.5% position tolerance and 1% band optimization.

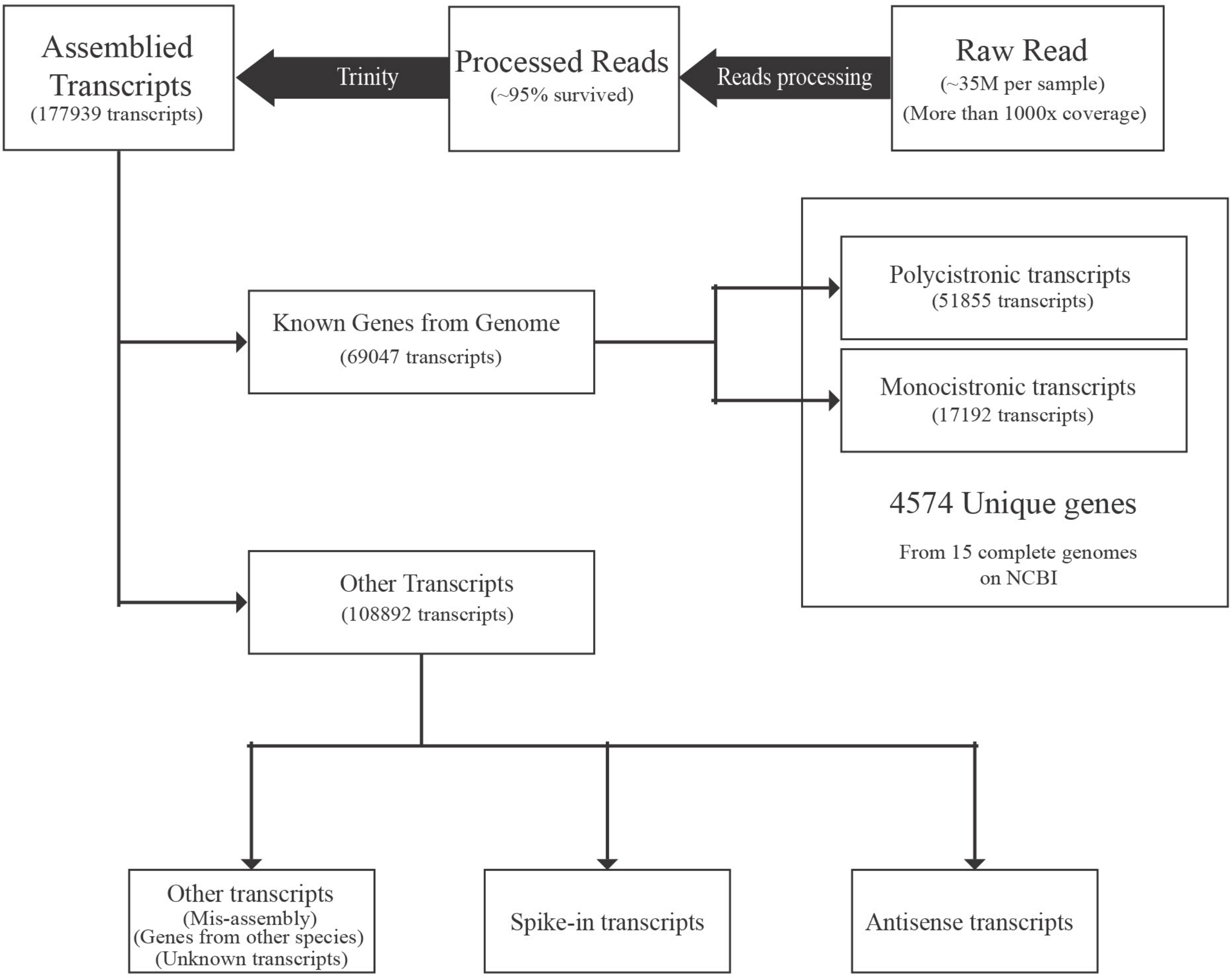

Figure S2. Bioinformatics analysis flow chart.

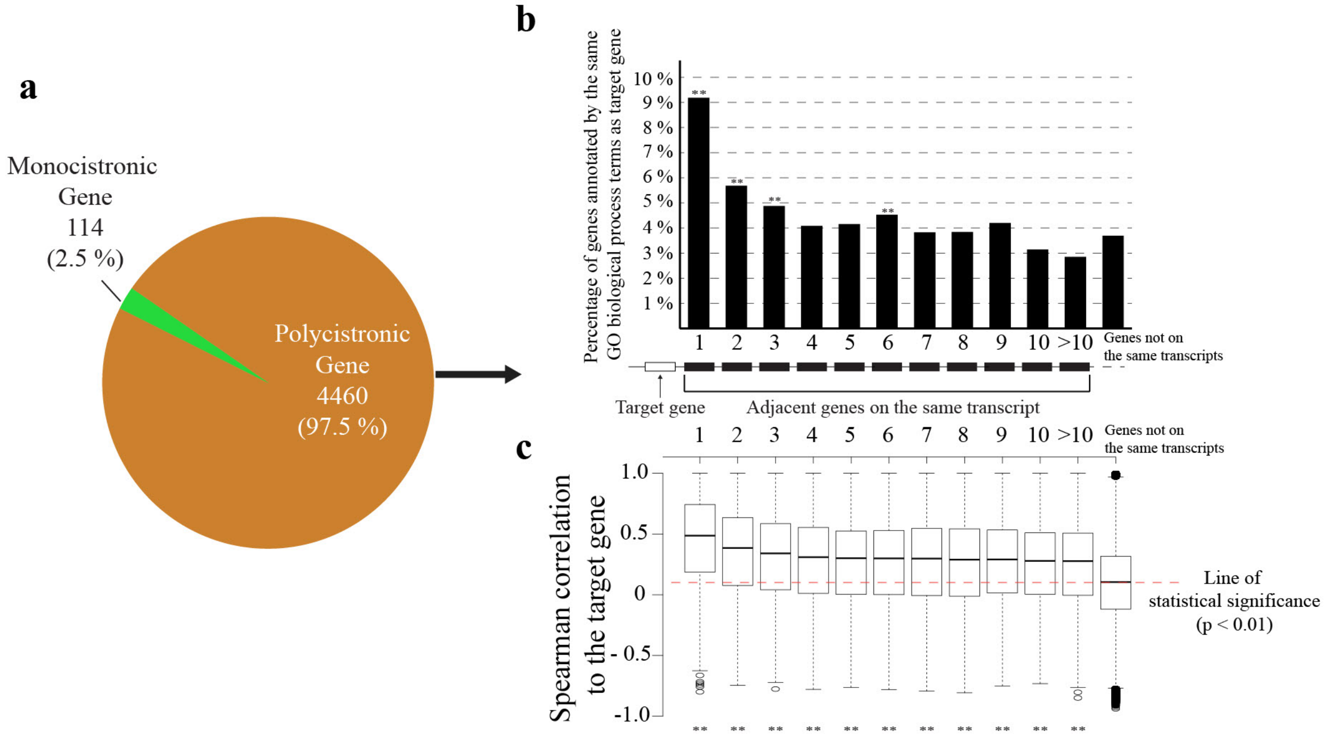

Figure S3. *De novo* assembly could efficiently construct polycistronic transcripts. (a) Distribution of genes being either monocistronic or polycistronic. (b) Proportion of adjacent genes which can be annotated by the same GO (biological process) as the target gene on the same polycistronic transcripts. (c) Boxplots of spearman correlations of adjacent genes with the target gene on the same polycistronic transcripts.

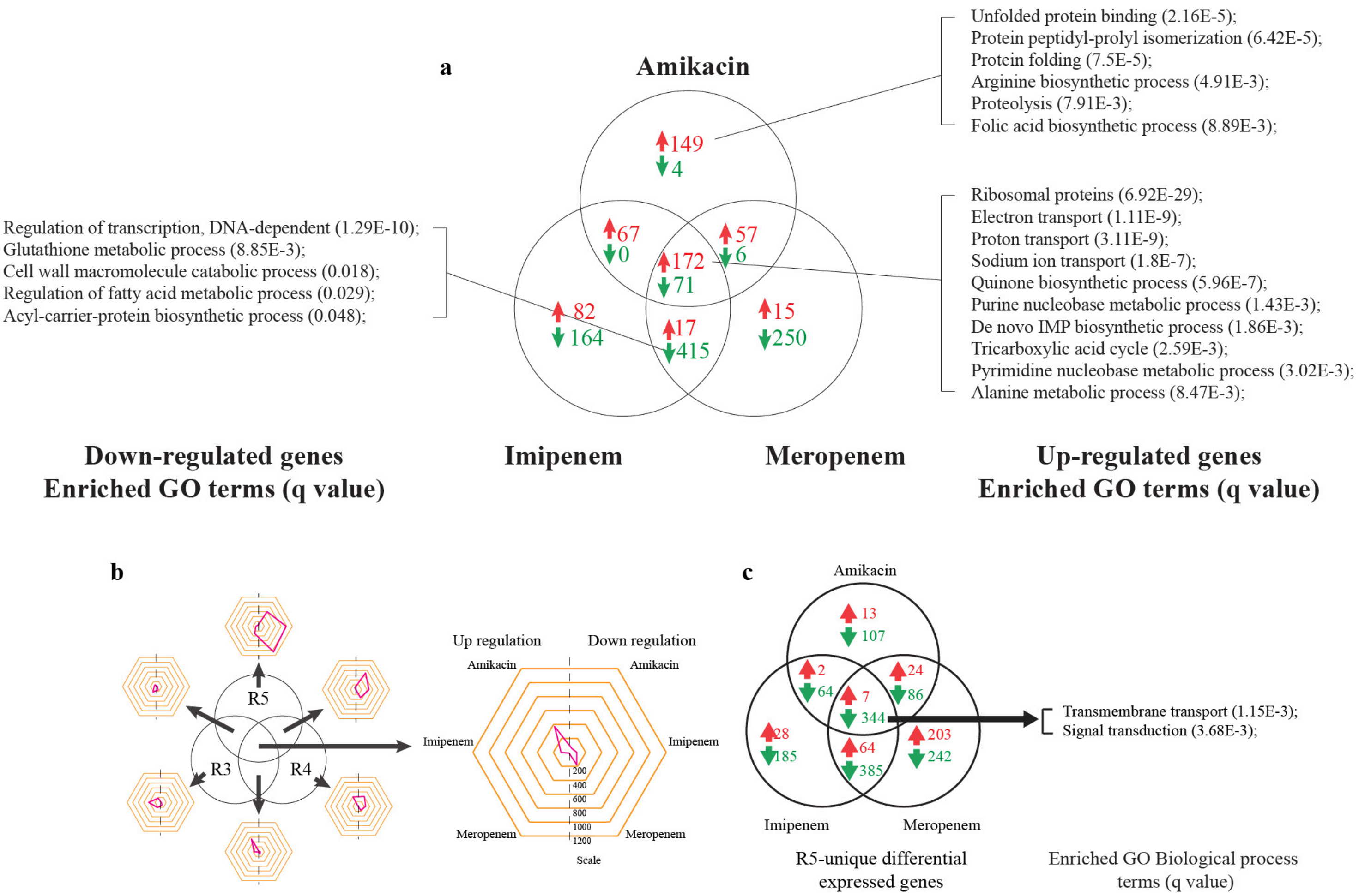

Figure S4. The three multidrug resistant strains showed differential responses under different antibiotic treatments. (a) Venn diagram and gene enrichment analysis of differentially-expressed genes under the three antibiotic treatments revealed both common and differential responses. (b) Venn diagram of differentially-expressed genes under antibiotic treatments in each strain. Scales and dimensions of all radar plots are the same as the middle example, which represents the common differentially-expressed genes among the three strains. (c) Venn diagram and gene enrichment analysis of differentially-expressed genes in R5 under the three antibiotic treatments.

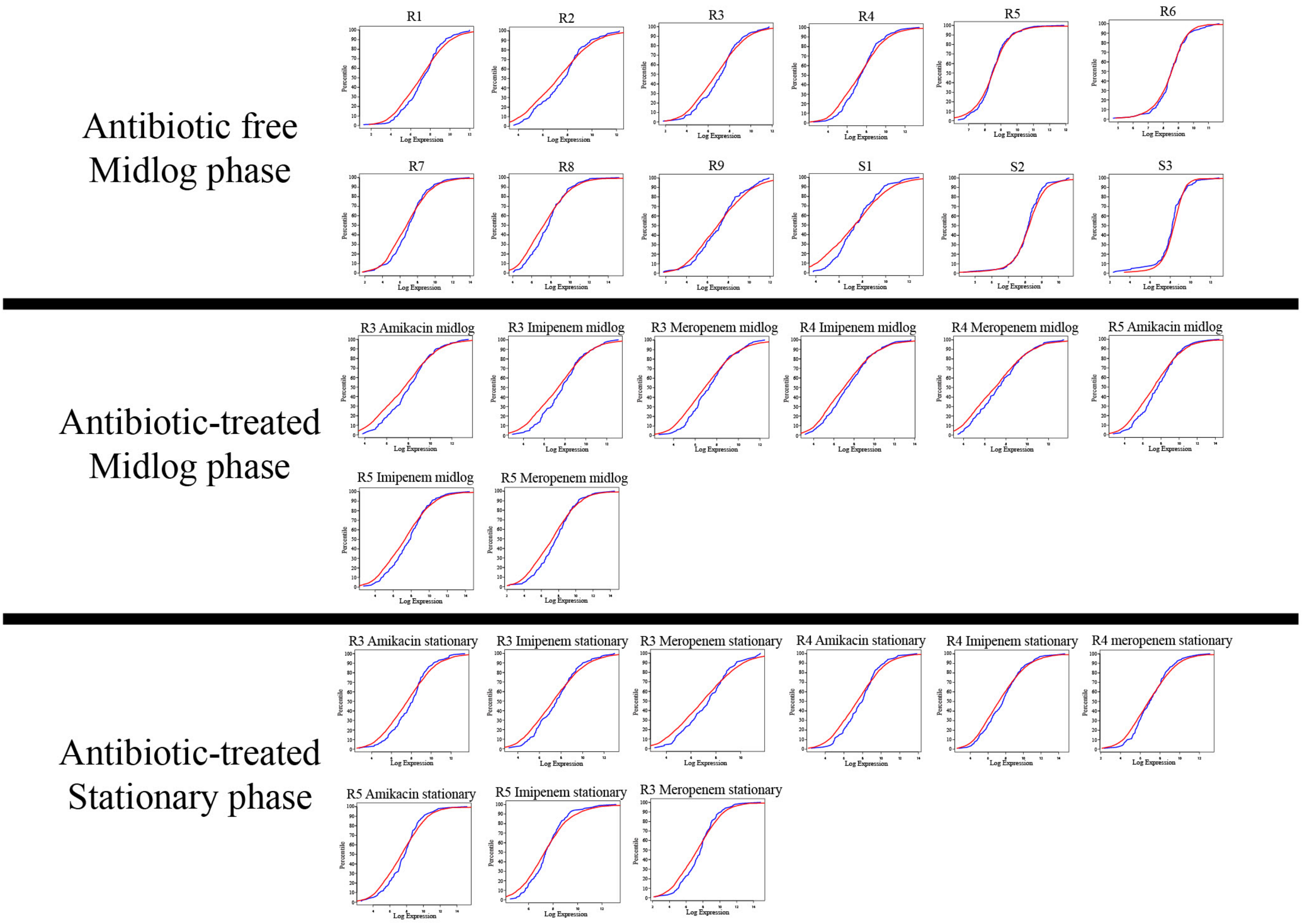

Figure S5. Comparison between transposon-associated and non-transposon-associated genes based on percentile of expression values across individual sample pairs. Blue line represents transposon-associated genes; red line represents non-transposon-associated genes.

a

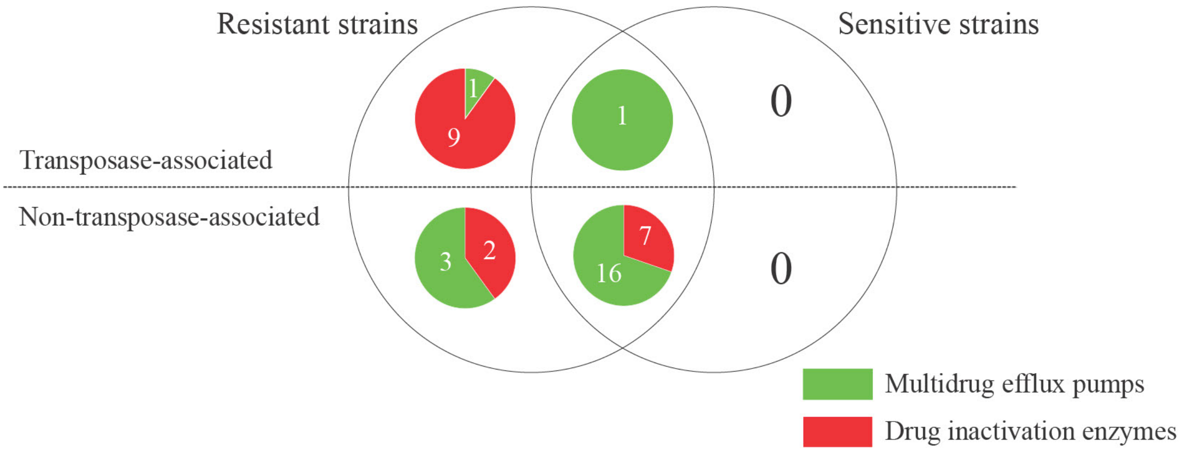

b

	Drug inactivation enzymes	Multidrug efflux pumps
Tranposase associated	9	2
Non-transposase associated	9	19

p = 0.005

c

	Similar expression in both groups	Only lowly-expressed in sensitive strains
Tranposase associated	1	10
Non-transposase associated	23	5

p = 0.000

d

	Similar expression in both groups	Only lowly-expressed in sensitive strains
Drug inactivation enzymes	7	11
Multidrug efflux pumps	17	4

p = 0.007

Figure S6. Transposon-associated resistant genes were more likely to be specific to multidrug resistant strains and co-transcribed with drug-inactivation enzymes. (a) Venn diagram of resistant genes. (b-d) Contingency table analysis of resistant genes' properties between: transposon-association vs. functions (b), transposon-association vs. expression patterns (c) and functions vs. expression patterns (d) p-values were calculated by Pearson's chi-square test.

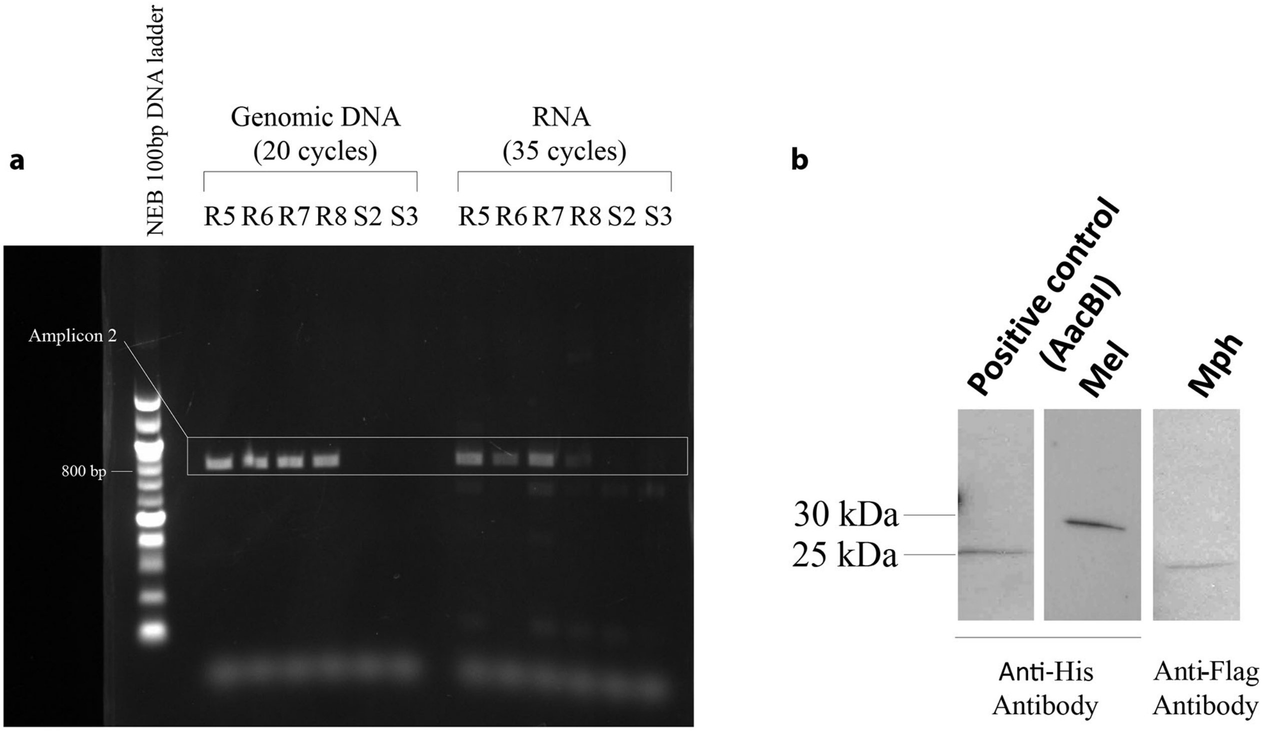

Figure S7. Expression validation of two antibiotic resistance genes in RNA and protein levels. (a) Weak co-transcription between tnpD and mel can be detected by qPCR after 35 cycles. (b) Western blotting confirmed the expression of Mel and Mph in plasmid-transformed cells.

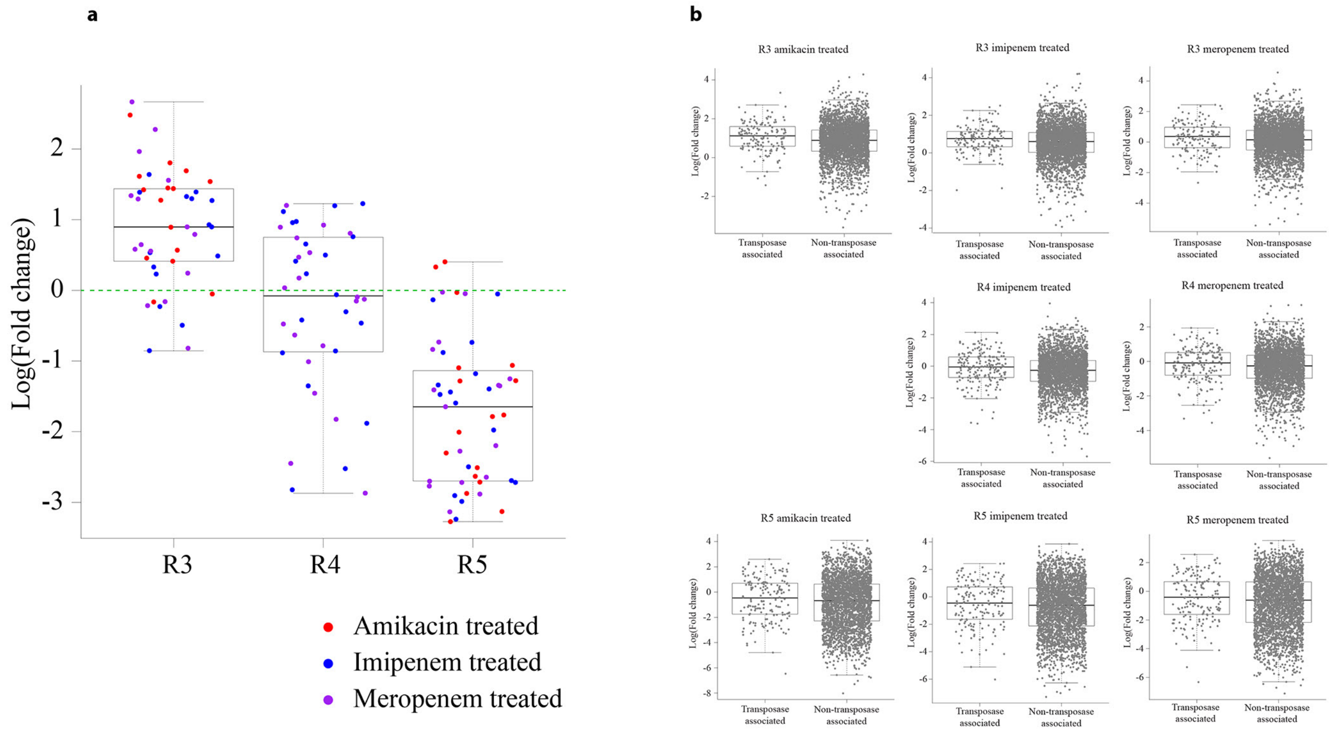

Figure S8. Expression patterns of transposable elements and transposon-associated genes. (a) Expressions patterns of transposable elements were strain-specific under antibiotic treatment. Each point represents a log(fold change) value of a transposable element gene under a certain antibiotic treatment. (b) Transposon-associated genes generally have higher fold changes under antibiotic treatment. Each point represents a log(fold change) value of a gene under a certain antibiotic treatment.
