# Supplementary material for: Comparative transcriptomics of multidrug-resistant Acinetobacter baumannii in response to antibiotic treatments
Source: Sci Rep. 2018 Feb 23;8:3515. doi: 10.1038/s41598-018-21841-9 (PMC5824817; doi:10.1038/s41598-018-21841-9)
Supplement: Supplementary file 3 — Supplementary tables 1–7 [file 41598_2018_21841_MOESM3_ESM.pdf]

Comparative transcriptomics of multidrug-resistant *Acinetobacter baumannii* in response to antibiotic treatments

Hao Qin[1,2]*, Norman Wai-Sing Lo[3], Jacky Fong-Chuen Loo[1], Xiao Lin[1,2], Aldrin Kay-Yuen Yim[1,2]*, Stephen Kwok-Wing Tsui[4], Terrence Chi-Kong Lau[5], Margaret Ip[3], and Ting-Fung Chan[1,2]#

[1]School of Life Sciences, The Chinese University of Hong Kong, Hong Kong SAR, China

[2]Partner State Key Laboratory of Agrobiotechnology, The Chinese University of Hong Kong, Hong Kong SAR, China

[3]Department of Microbiology, The Chinese University of Hong Kong, Hong Kong SAR, China

[4]School of Biomedical Sciences, The Chinese University of Hong Kong, Hong Kong SAR, China

[5]Department of Biomedical Sciences, City University of Hong Kong, Hong Kong SAR, China

#Address correspondence to Ting-Fung Chan, tf.chan@cuhk.edu.hk.

*Present address: Hao Qin, 3D Medicines Corporation, Shang Hai, China; Aldrin Kay-Yuen Yim, Washington University School of Medicine, Saint Louis, MO, USA.

N.W.S.L and J.F.C.L contributed equally to this work.

Supplementary Tables

Table S1. Minimum inhibitory concentrations (MICs) of strains used in this study.

	Minimum inhibitory concentration (MIC) μg/ml										
Strains	AMK	FEP	CFP-SUL	CAZ	CIP	GEN	IPM	LVX	MEM	PIP	TZP
R1	256 (R)	128 (R)	32	128 (R)	64 (R)	>128 (R)	32 (R)	4 (I)	64 (R)	512 (R)	>128 (R)
R2	4 (S)	128 (R)	32	128 (R)	64 (R)	128 (R)	16 (R)	4 (I)	4 (S)	512 (R)	>128 (R)
R3	2 (S)	>128 (R)	32	128 (R)	64 (R)	64 (R)	32 (R)	4 (I)	4 (S)	512 (R)	128 (R)
R4	>256 (R)	64 (R)	32	64 (R)	64 (R)	>128 (R)	2 (S)	8 (R)	2 (S)	512 (R)	128 (R)
R5	>256 (R)	64 (R)	64	128 (R)	32 (R)	>128 (R)	>32 (R)	4 (I)	>64 (R)	>512 (R)	>128 (R)
R6	>256 (R)	>128 (R)	64	>128 (R)	64 (R)	>128 (R)	>32 (R)	4 (I)	64 (R)	>512 (R)	>128 (R)
R7	>256 (R)	128 (R)	32	128 (R)	64 (R)	>128 (R)	>32 (R)	8 (R)	>64 (R)	>512 (R)	>128 (R)
R8	>256 (R)	64 (R)	32	64 (R)	32 (R)	>128 (R)	32 (R)	4 (I)	32 (R)	>512 (R)	>128 (R)
R9	2 (S)	128 (R)	32	16 (I)	16 (R)	2 (S)	>32 (R)	8 (R)	32 (R)	512 (R)	>128 (R)
S1	2 (S)	4 (S)	2	4 (S)	0.25 (S)	0.5 (S)	0.25 (S)	0.12 (S)	0.25 (S)	16 (S)	16 (S)
S2	2 (S)	4 (S)	32	2 (S)	0.12 (S)	0.5 (S)	0.12 (S)	0.06 (S)	0.25 (S)	16 (S)	2 (S)
S3	2 (S)	8 (S)	2	4 (S)	0.12 (S)	0.5 (S)	0.12 (S)	≤0.03 (S)	0.12 (S)	8 (S)	8 (S)

Antibiotics abbreviations: AMK: Amikacin; FEP: Cefepime; CFP-SUL: Cefoperazone-sulbactam; CAZ: Ceftazidime; CIP: Ciprofloxacin; GEN: Gentamicin; IPM: Imipenem; LVX: Levofloxacin; MEM: Meropenem; PIP: Piperacillin; TZP: Piperacillin/tazobactam.

(R): Resistant; (I): Intermediate resistant; (S): Sensitive.

Table S2. Strain information.

Strain name	Isolation Site	PFGE Pattern	MLST (Oxford)							
			gltA	gyrB	gdhB	recA	cpn60	gpi	rpoD	ST
R1	MSU	Ia	1	3	3	2	2	97	3	208
R2	Bag Urine	Ia	1	3	3	2	2	97	3	208
R3	PCN Urine	Ia	1	3	3	2	2	97	3	208
R4	Sputum	Ia	1	3	3	2	2	97	3	208
R5	Blood Culture	Ia	1	3	3	2	2	102	3	218
R6	Wound Swab	Ia	1	3	3	2	2	96	3	195
R7	Blood Culture	Ia	1	3	3	2	2	102	3	218
R8	Wound Swab	Ib	21	15	3	2	35	202	4	806
R9	Bag Urine	II	1	43	50	31	1	94	26	940
S1	Blood Culture	V	21	12	2	28	1	157	4	new
S2	Blood Culture	III	1	96	11	48	18	114	43	new
S3	Blood Culture	II	1	15	56	1	1	187	45	new

Table S3. Plasmid constructions for transformation.

Gene name	Plasmid construct	Plasmid construction method	Tag
Mel	pET101-TOPO-Mel	Champion™ pET101 Directional TOPO® Expression Kit	V5 isotope, 6x His tag
Mph	pET15b-Mph	Gene synthesis	Flag tag
Positive control AacBI	pET101-TOPO-AacBI	Champion™ pET101 Directional TOPO® Expression Kit	V5 isotope, 6x His tag
Negative control	pET101-TOPO-negative	Champion™ pET101 Directional TOPO® Expression Kit	NA

Table S4. Antibiotic concentrations of applied for the transcriptome study.

Strain	Antibiotics	Concentration (μg/ml)	Proportion of MIC
R3	Amikacin	0.03125	1/64
	Imipenem	1	1/32
	Meropenem	0.5	1/8
R4	Amikacin	4	1/64
	Imipenem	0.0625	1/32
	Meropenem	0.25	1/8
R5	Amikacin	4	1/64
	Imipenem	1	1/32
	Meropenem	8	1/8

Table S5. Published genomes used in this study.

Strain name	NCBI Accession ID
ATCC 17978	NC_009085.1
1656-2	NC_017162.1
AB0057	NC_011586.1
AB307-0294	NC_011595.1
ACICU	NC_010611.1
AYE	NC_010410.1
BJAB07104	NC_021726.1
BJAB0715	NC_021733.1
BJAB0868	NC_021729.1
D1279779	NC_020547.1
MDR-TJ	NC_017847.1
MDR-ZJ06	NC_017171.1
SDF	NC_010400.1
TCDC-AB0715	NC_017387.1
TYTH-1	NC_018706.1

Table S6. Primers used in this study.

Gene id	Gene	Annealing temperature	Amplicon size	Forward primer	Reverse primer
**PCR**					
G2819, G2828, G2826	Amikacin-resistant candidate region amplicon 1	57 ^{O}C	3951 nt	TCCTCACCTTTGTCACGCC	CCCAACTGAGCTTTTGCTCC
G2819, G2826	Amikacin-resistant candidate region amplicon 2	57 ^{O}C	795 nt	TGCAAAGAACCCAAACAGCG	ACATTGGAAACACCAAGGCG
G2828, G2826	Amikacin-resistant candidate region amplicon 3	57 ^{O}C	453 nt	GAGTGCGAGTACACAGTTGC	TTGTCCGTAAGCAACAAGCC
**qPCR**					
G2819	Transposase tnpD	60 ^{O}C	141 nt	CAATGCCGATACTGCCAAGC	AGCTGCTTTAAAAACGCCCC
G2828	Macrolide 2'-phosphotransferase	60 ^{O}C	134 nt	ATCACTTGCTGAAGCACACG	CATCACGACGAGGAATACGC
G2826	Macrolide efflux protein	60 ^{O}C	82 nt	AAACGCCAGCAAGCTAATCG	CAAGTCGTCCAGCACTTTCG
**Cloning**					
G2826	Macrolide efflux protein (Mel)	55 ^{O}C	1473 nt	CACCATGAGTTTAATTATTAAAGCGAGAAACATACG	ACAATCTCTCTCAAAAGTCTTGATGA
G1418	Aminoglycoside 6'-N-acetyl transferase type Ib (AacBI)	62 ^{O}C	698 nt	CACCGCCTCGGGCATCCAAGCAG	GGCAACACTGCGTGTTCGCT

Table S7. Transposon-resistance gene combinations upregulated under antibiotic treatment

Antibiotic resistant gene		Associated transposon	
G2966	beta-lactamase OXA-23	G2278	transposition helper
G3868	streptomycin 3''-kinase	G3891	hypothetical protein
		G3584	transposase 1
		G3585	transposase
		G3902	transposase
G4338	Gentamicin acetyltransferase I (AAC(3)-I)	G4252	transposase
		G4274	transposase
G4241	beta-lactamase TEM	G4252	transposase
		G4274	transposase
		G4285	Site-specific recombinase, DNA invertase Pin-like protein
G2792	Chloramphenicol acetyltransferase	G4252	transposase
		G4274	transposase
		G4285	recombinase, DNA invertase Pin-like protein
G3879	Streptomycin 6-kinase	G3891	hypothetical protein
		G3902	transposase
		G3584	transposase 1
		G3585	transposase
G4342	Aminoglycoside adenyltransferase	G4252	transposase
		G4274	transposase
		G4285	recombinase, DNA invertase Pin-like protein
G1418	aminoglycoside 6'-N-acetyl transferase type Ib	G4252	transposase
		G4274	transposase
		G4285	recombinase, DNA invertase Pin-like protein
G2790	aminoglycoside 3'-phosphotransferase aphA1	G4252	transposase
		G4274	transposase
		G4285	recombinase, DNA invertase Pin-like protein
